# Trends in emotional distress among childhood, adolescent, and young adult (CAYA) cancer survivors: A decade-long study

**DOI:** 10.1007/s00520-026-10580-7

**Published:** 2026-03-23

**Authors:** Pranali G. Patel, Chaitali S. Dagli, Abdulghafoor Alani, Mrudula Nair, Nada Al-Antary, Oluwole A. Babatunde, Dina K. Abouelella, Nosayaba Osazuwa-Peters, Eric Adjei Boakye

**Affiliations:** 1 Department of Epidemiology, University of Alabama at Birmingham, Birmingham, AL USA; 2 Central Michigan University College of Medicine, Saginaw, MI USA; 3 Department of Public Health Sciences, Henry Ford Health, One Ford Place, Detroit, MI 48202 USA; 4 Prisma Health, Greer, SC, USA; 5 Department of Head and Neck Surgery & Communication Sciences, Duke University School of Medicine, Durham, NC USA; 6 Duke Cancer Institute, Duke University, Durham, NC USA; 7 Department of Population Health Sciences, Duke University School of Medicine, Durham, NC USA; 8 Department of Otolaryngology—Head and Neck Surgery, Henry Ford Health, Detroit, MI USA; 9 Henry Ford Health + Michigan State University Health Sciences, Detroit, MI USA; 10 Department of Epidemiology and Biostatistics, Michigan State University College of Human Medicine, East Lansing, MI USA

**Keywords:** Childhood cancers, AYA cancers, Cancer survivors, Psychological distress, Mental health, Emotional distress

## Abstract

**Purpose:**

We examined trends in emotional distress among survivors of childhood and adolescent and young adult (CAYA) cancers.

**Methods:**

We analyzed the 2008–2018 National Health Interview Survey (NHIS) data among individuals (*n* = 6451) who were diagnosed with cancer between 0 and 39 years of age. Emotional distress was assessed using the validated Kessler 6-item scale. Respondents rated how often they felt nervous, hopeless, restless, or fidgety, so sad that nothing could cheer them up, that everything was an effort, and worthless in the past 30 days. Responses were scored and added to produce a range of 0–24. We classified emotional distress as mild/no (score of less <5), moderate (score between 5 and 12), or severe distress (≥ 13). Joinpoint regression estimated yearly increases/decreases in psychological distress using annual percent changes.

**Results:**

Overall, the proportion of individuals experiencing mild/no psychological distress increased by 1.68% annually between 2008 and 2014 and then decreased by 1.34% annually between 2014 and 2018, although not statistically significant. The proportion of individuals experiencing moderate distress decreased by 3.06% annually between 2008 and 2014 and then increased by 4.18% annually between 2014 and 2018, although not statistically significant. The proportion of individuals experiencing severe distress remained stable between 2008 and 2014 and then decreased by 7.36% annually between 2014 and 2018, although not statistically significant. No statistically significant trend in emotional distress was observed when stratified by patients’ demographics and access to mental health services.

**Conclusion:**

We found that trends in emotional distress among survivors of CAYA cancers have not changed significantly over the 2008–2018 decade, overall and when stratified by gender, race/ethnicity, marital status, and visit to a mental health professional within the past year.

**Supplementary Information:**

The online version contains supplementary material available at 10.1007/s00520-026-10580-7.

## Introduction

There were approximately 18.1 million cancer survivors in the United States (U.S.) as of January 2022 [1], representing 5.4% of the total U.S. population. By 2040, the number of cancer survivors is projected to reach 26 million [2]. The population of cancer survivors diagnosed at childhood or adolescent and young adult (CAYA) age is currently estimated at 400,000 in the U.S., and it  is steadily increasing [2–5]. In addition, with financial burden of an estimated $279,648 per childhood cancer survivor [6] and $259,324 per adolescent and young adult survivor [7], survivors of CAYA cancer encounter unique challenges.

Navigating the critical transition from adolescence to young adulthood is already psychologically demanding [8]. However, for individuals diagnosed with cancer during this stage, the journey becomes even more complex due to the added physical and emotional challenges of the disease and its treatment. Cancer survivors often experience chronic pain, which is linked to worsened mental health, functionality, and employment outcomes [9]. Recent studies have shown that childhood cancer survivors have more frequent mental health visits [10], a higher prevalence of emotional distress symptoms [11, 12], cognitive impairment, physical disability, and chronic health issues [13]. Adolescent and young adult (AYA) cancer survivors also face a wide range of physical and psychological late effects that significantly impact their quality of life [14]. Compared to survivors diagnosed in middle or older adulthood, survivors diagnosed as AYAs experience a greater prevalence of emotional distress [12]. Another study found that 15.1% of childhood cancer survivors reported experiencing elevated levels of overall emotional distress [13].

However, these studies, being cross-sectional, cannot capture changes in emotional distress over the course of survivorship. Longitudinal studies on childhood cancer survivors indicate that most survivors report minimal to no distress, with elevated symptoms affecting only a specific group, aligning with the concept of post-traumatic growth [15, 16]. Limited research has explored the changes in emotional distress over time among survivors of CAYA cancers. This study aims to examine trends in emotional distress experienced by adult survivors of CAYA cancers between 2008 and 2018 in the U.S. By employing trend analysis, we will identify factors that have changed over time with respect to emotional distress. Understanding these patterns is crucial for optimizing the timing of screening measures and interventions; ultimately helping CAYA survivors manage their distress during critical post-treatment phases, and addressing overlapping challenges and improving quality of life.

## Methods

### Data source

We examined data from 2008 to 2018 National Health Interview Survey (NHIS), which is conducted annually by the National Center for Health Statistics, Centers for Disease Control and Prevention [17]. NHIS is a cross-sectional household interview survey of the civilian noninstitutionalized population residing in the United States at the time of the interview. NHIS is used to monitor the health of the United States population through the collection and analysis of data on a broad range of health topics. NHIS interviews are completed in person in participants’ homes. The sample design is a probability design that permits the representative sampling of households and noninstitutional group quarters and oversamples Hispanic and Black populations. The sample adult survey component, which includes information on health conditions for adults 18 years old or older, was used in this study. Because these data are publicly available, our study did not require an IRB approval and informed consent.

### Study cohort

Our study sample was restricted to participants who were diagnosed with cancer when they were 0 to 39 years old. Cancer survivors were identified using the NHIS question that asked about ever being told by a physician or health professional that she/he had cancer or a malignancy of any kind. Nonmelanoma skin cancer or skin cancer with unknown type were excluded from the analytical sample.

### Outcome variable

The outcome variable was psychological distress, measured by the validated Kessler 6 (K6) nonspecific distress scale of six symptoms [18]. Respondents were asked how often they felt the following in the past 30 days: 1) “So sad nothing cheers them up”, 2) “nervous”, 3) “restless/fidgety”, 4) “hopeless”, 5) “everything was an effort”, and 6) “worthless”. The responses were on a 5-point Likert scale which were scored as 0 — “None of the time”, 1 — “A little of the time”, 2 — “Some of the time”, 3 — “Most of the time”, 4 — “All of the time”. Responses were scored and added to produce a range of 0–24. We further categorized the score into three levels based on previous literature with values of 0–4 being classified as mild distress, 5–12 as moderate distress, and ≥ 13 as severe psychological distress [12].

### Covariates

Trend analyses were stratified by gender, race/ethnicity, marital status, and visit to mental health professional within past year. Marital status was categorized as “currently married/living with partner”, “widowed/divorced/separated”, and “never married”. Race/Ethnicity was categorized as “non-Hispanic White”, “non-Hispanic Black”, and “Hispanic”. Visit to a mental health professional within past 12 months was classified as “yes” or “no”.

### Statistical analyses

Analyses were weighted to account for the complex survey design of the NHIS that reduced bias owing to nonresponse and noncoverage and allowed the results to be generalized to the U.S. adult population. Descriptive statistics using Chi square test were performed to describe the cohort characteristics and stratified by psychological distress. We reported frequency and percentages for all categorical variables. Trends in psychological distress were calculated using joinpoint regression, a variant of log-linear regression [19]. Joinpoint regression models determined the starting and ending years of increases/decreases and then estimated the annual percentage change (APC) and 95% confidence intervals (CI) on the basis of regression model between the 2 joinpoint years [20]. The final joinpoint models were based on log-transformed percentages to better ensure the normality of residuals. The permutation test method determined the model with the fewest number of joinpoints necessary to effectively characterize trends with a maximum of 2 joinpoints selected in this study. Tests for the model selection and significant increases/decreases in APCs were set at alpha = 0.05 and all tests were two-tailed. Joinpoint regression was performed in Joinpoint 4.9.0.1 (National Cancer Institute Statistical Research Applications Branch, Bethesda, MD). Descriptive statistics were conducted by using SAS, version 9.4 (SAS Institute, Inc).

## Results

There were 6451 survey respondents in our study between years 2008 and 2018. Overall, 8% of respondents reported experiencing severe distress, and 26% reported moderate distress. Respondents were 43.9% aged 18–44, 71.5% female, 82.3% non-Hispanic White, and 62.5% married or living as married. Approximately 30.9% had a college degree or more, and 85.3% had not seen a mental health professional. Finally, 38.2% resided in the South region of the U.S. **(**Table 1**)**.

**Table 1:** Sample characteristics overall and stratified by emotional distress, 2008–2018 National Health Interview Survey (*n* = 6451)

	**Frequency (weighted percent)**				***P*-value**
	**Total (100%)**	***Emotional distress***			
		**Low/None (66.3%)**	**Moderate (25.6%)**	**Severe (8.1%)**	
**Age at time of survey**					<0.0001*
18–44	2634 (43.9)	1575 (62.2)	761 (28.4)	251 (9.4)	
45–64	2441 (38.8)	1524 (66.5)	637 (25.3)	238 (8.2)	
65+	1340 (17.3)	993 (76.3)	258 (19.0)	60 (4.7)	
**Sex**					<0.0001*
Male	1677 (28.5)	1197 (73.0)	354 (21.7)	95 (5.3)	
Female	4738 (71.5)	2895 (63.7)	1302 (27.1)	454 (9.3)	
**Race/ethnicity**					0.1066
Non-Hispanic White	5058 (82.3)	3295 (67.0)	1274 (25.3)	400 (7.7)	
Non-Hispanic Black	520 (6.5)	299 (63.6)	149 (27.0)	57 (9.4)	
Hispanic	619 (8.4)	363 (61.3)	171 (27.1)	75 (11.7)	
Missing	218 (2.9)	135 (67.9)	62 (25.2)	17 (6.9)	
**Marital status**					<0.0001*
Married/Living as Married	3213 (62.5)	2226 (70.7)	741 (23.4)	199 (5.9)	
Divorced/Widowed/Separated	2151 (23.6)	1252 (58.6)	596 (28.7)	259 (12.7)	
Never Married	1051 (13.9)	614 (59.6)	319 (29.9)	91 (10.6)	
**Education level**					
Less than High School	810 (11.6)	377 (48.7)	258 (32.0)	153 (19.3)	<0.0001*
High School Graduate	1463 (23.4)	867 (59.8)	420 (30.1)	150 (10.1)	
College Graduate or More	1850 (30.9)	1415 (78.0)	354 (18.9)	50 (3.1)	
Some College	2274 (33.7)	1425 (66.4)	615 (26.0)	195 (7.6)	
Missing	18 (0.4)	8 (34.9)	9 (57.4)	1 (7.7)	
**Visit mental health professional past 12 months**					
Yes	943 (13.5)	316 (35.0)	403 (43.2)	210 (21.9)	<0.0001*
No	5393 (85.3)	3753 (71.2)	1246 (22.8)	336 (6.0)	
Missing	79 (1.2)	23 (78.3)	7 (16.0)	3 (5.6)	
**Geographic region**					
Northeast	896 (14.6)	589 (68.4)	220 (23.8)	74 (7.8)	0.6971
Midwest	1539 (24.4)	964 (64.1)	402 (26.8)	137 (9.1)	
South	2303 (38.2)	1471 (66.7)	589 (25.5)	201 (7.8)	
West	1677 (22.8)	1068 (66.6)	445 (25.5)	137 (7.9)	
**Survey year**					0.1602
2008	444 (9.5)	258 (61.3)	131 (28.4)	48 (10.4)	
2009	519 (8.6)	328 (63.9)	141 (27.7)	49 (8.5)	
2010	504 (9.1)	329 (67.6)	133 (24.7)	37 (7.7)	
2011	591 (8.6)	381 (66.1)	156 (25.4)	52 (8.5)	
2012	646 (9.0)	425 (67.2)	165 (25.0)	55 (7.8)	
2013	649 (9.2)	392 (64.0)	180 (27.9)	59 (8.1)	
2014	715 (9.2)	474 (73.2)	160 (19.5)	62 (7.3)	
2015	636 (8.8)	392 (66.1)	152 (23.4)	61 (10.5)	
2016	650 (9.1)	422 (67.1)	155 (24.8)	58 (8.2)	
2017	562 (9.8)	358 (65.1)	160 (29.7)	32 (5.2)	
2018	499 (9.1)	333 (68.0)	123 (24.4)	36 (7.6)	

*p-value <0.05 as test of significance

**Fig. 1:**
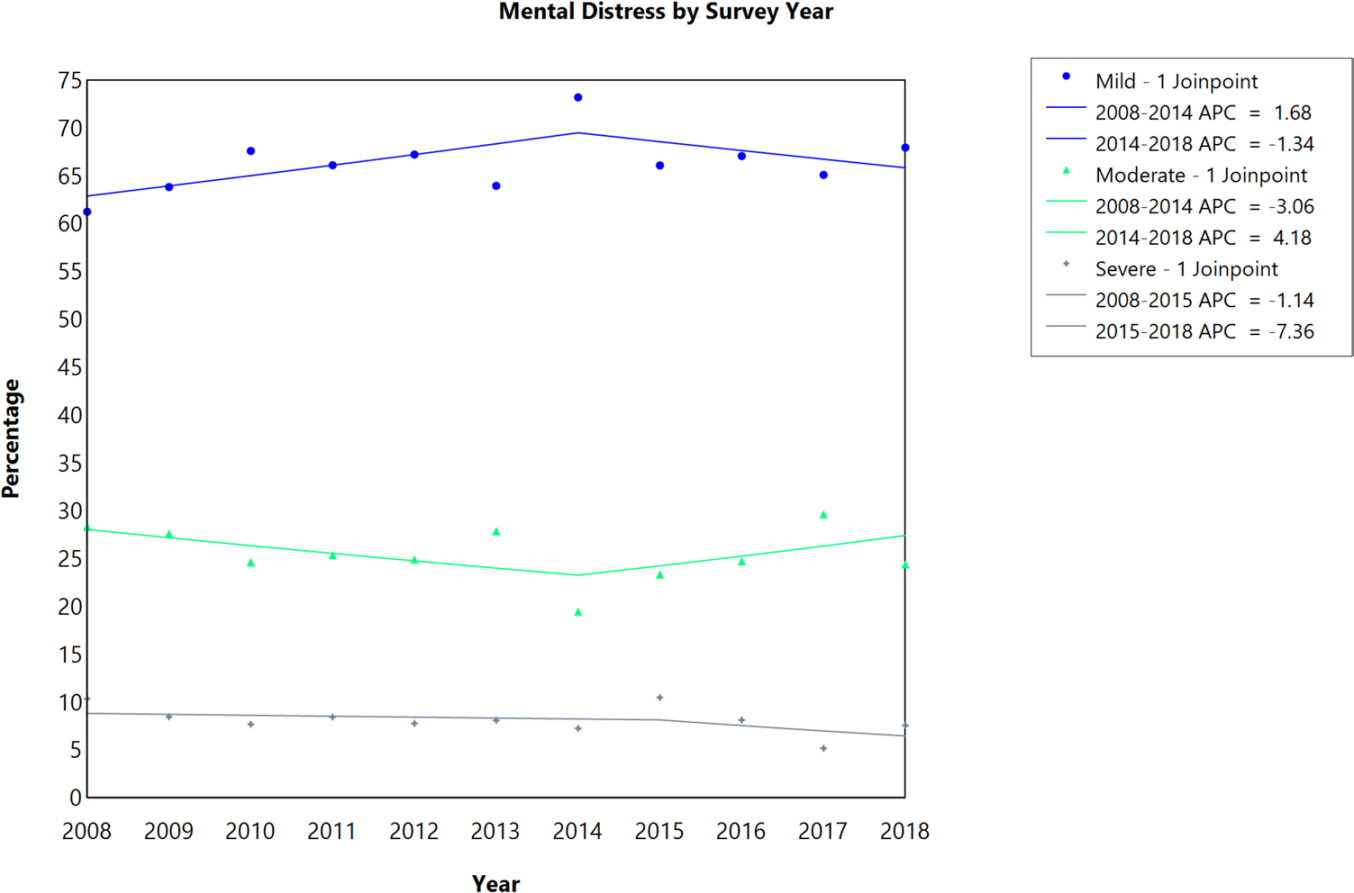
Joinpoint analysis of the prevalence of emotional distress among cancer survivors diagnosed at childhood or adolescent and young adult, 2008–2018 National Health Interview Survey. APC, annual percentage change; ^*^P-value < 0.05 as test of significance. Data: 2008–2018 National Health Interview Survey

Overall, the proportion of individuals experiencing mild/no psychological distress increased by 1.68% annually between 2008 and 2014 (APC = 1.68; 95% CI: −0.35 to 3.75) and then decreased by 1.34% annually between 2014 and 2018 (APC = −1.34; 95% CI: −4.77 to 2.21); however, the trends were not statistically significant (Fig. 1, Table 2). In contrast, the proportion of individuals experiencing moderate distress decreased by 3.06% annually between 2008 and 2014 (APC = −3.06; 95% CI: −8.12 to 2.29) and then increased by 4.18% annually between 2014 and 2018 (APC = 4.18; 95% CI: −6.53 to 16.12), and these trends were not statistically significant (Fig. 1, Table 2). The proportion of individuals experiencing severe distress decreased by 1.14% annually between 2008 and 2014 (APC = −1.14; 95% CI: −7.48 to 5.63) and then decreased by 7.36% annually between 2014 and 2018 (APC = −7.36; 95% CI: −28.56 to 20.15); a non-significant trend remained (Fig. 1, Table 2).

**Table 2:** Annual percentage change (APC) over time in emotional distress, 2008–2018 National Health Interview Survey

	**Years**	**APC**	**Lower CI**	**Upper CI**	***P*-value**
**Overall trend**					
Mild	2008–2014	1.68	−0.35	3.75	0.090
Mild	2014–2018	−1.34	−4.77	2.21	0.386
Moderate	2008–2014	−3.06	−8.12	2.29	0.206
Moderate	2014–2018	4.18	−6.53	16.12	0.391
Severe	2008–2015	−1.14	−7.48	5.63	0.687
Severe	2015–2018	−7.36	−28.56	20.15	0.499
**Sex**					
*Female*					
Mild	2008–2010	6.23	−14.80	32.46	0.528
Mild	2010–2018	−0.20	−2.25	1.89	0.820
Moderate	2008–2014	−3.11	−9.03	3.20	0.267
Moderate	2014–2018	4.04	−9.13	19.10	0.501
Severe	2008–2010	−12.41	−58.98	87.05	0.684
Severe	2010–2018	0.50	−8.17	9.98	0.897
*Male*					
Mild	2008–2014	1.28	−1.35	3.97	0.281
Mild	2014–2018	−1.27	−5.20	2.82	0.469
Moderate	2008–2014	−2.64	−12.14	7.89	0.548
Moderate	2014–2018	4.98	−11.45	24.47	0.511
Severe	2008–2010	13.56	−68.04	303.49	0.814
Severe	2010–2018	−8.42	−19.43	4.11	0.144
**Race/Ethnicity**					
*Non-Hispanic White*					
Mild	2008–2014	1.95	−0.39	4.35	0.088
Mild	2014–2018	−2.07	−6.08	2.10	0.265
Moderate	2008–2014	−3.55	−9.58	2.89	0.220
Moderate	2014–2018	5.83	−7.03	20.46	0.326
Severe	2008–2015	0.18	−7.23	8.17	0.957
Severe	2015–2018	−9.79	−32.60	20.74	0.420
*Non-Hispanic Black*					
Mild	2008–2011	13.58	−12.37	47.22	0.275
Mild	2011–2018	1.22	−3.76	6.47	0.577
Moderate	2008–2014	−8.72	−15.83	−1.01	0.033^*^
Moderate	2014–2018	4.45	−17.46	32.18	0.667
Severe	2008–2016	−11.80	−29.46	10.28	0.218
Severe	2016–2018	19.21	−96.04	3489.78	0.904
*Hispanic*					
Mild	2008–2012	4.99	−7.63	19.34	0.388
Mild	2012–2018	−2.51	−9.67	5.23	0.447
Moderate	2008–2010	13.11	−44.57	130.78	0.687
Moderate	2010–2018	−0.16	−7.49	7.76	0.961
Severe	2008–2010	−32.82	−88.68	298.77	0.604
Severe	2010–2018	2.13	−17.98	27.17	0.822
**Marital status**					
*Married*					
Mild	2008–2014	2.26	−0.47	17.38	0.058
Mild	2014–2018	−3.26	−15.28	0.78	0.068
Moderate	2008–2014	−4.19	−10.12	2.14	0.153
Moderate	2014–2018	7.34	−5.83	22.34	0.234
Severe	2008–2014	−4.12	−17.14	10.95	0.507
Severe	2014–2018	4.60	−20.07	36.88	0.697
*Divorced/Separated*					
Mild	2008–2015	0.48	−4.67	5.91	0.831
Mild	2015–2018	2.43	−12.77	20.27	0.727
Moderate	2008–2013	0.30	−15.27	18.73	0.967
Moderate	2013–2018	−4.65	−19.49	12.91	0.516
Severe	2008–2016	2.14	−8.72	14.29	0.661
Severe	2016–2018	−18.30	−75.14	168.54	0.692
*Never Married*					
Mild	2008–2010	12.56	−25.40	69.83	0.508
Mild	2010–2018	0.57	−2.73	3.98	0.691
Moderate	2008–2010	−15.51	−54.54	57.02	0.530
Moderate	2010–2018	2.83	−3.83	9.95	0.347
Severe	2008–2014	−2.50	−24.10	25.25	0.813
Severe	2014–2018	−29.76	−63.91	36.72	0.242
**Visit mental health professional past 12 months**					
*Yes*					
Mild	2008–2016	−1.06	−9.28	7.90	0.774
Mild	2016–2018	15.68	−41.23	127.72	0.618
Moderate	2008–2010	−3.84	−44.62	66.99	0.868
Moderate	2010–2018	1.32	−4.20	7.17	0.588
Severe	2008–2015	0.47	−10.50	12.78	0.925
Severe	2015–2018	−10.58	−42.17	38.25	0.553
*No*					
Mild	2008–2014	1.78	0.77	7.00	0.013*
Mild	2014–2018	−1.52	−6.43	0.28	0.108
Moderate	2008–2015	−3.26	−8.04	1.76	0.160
Moderate	2015–2018	7.52	−10.38	29.00	0.367
Severe	2008–2010	−12.92	−58.38	82.18	0.663
Severe	2010–2018	−1.27	−9.29	7.47	0.726

**Fig. 2:**
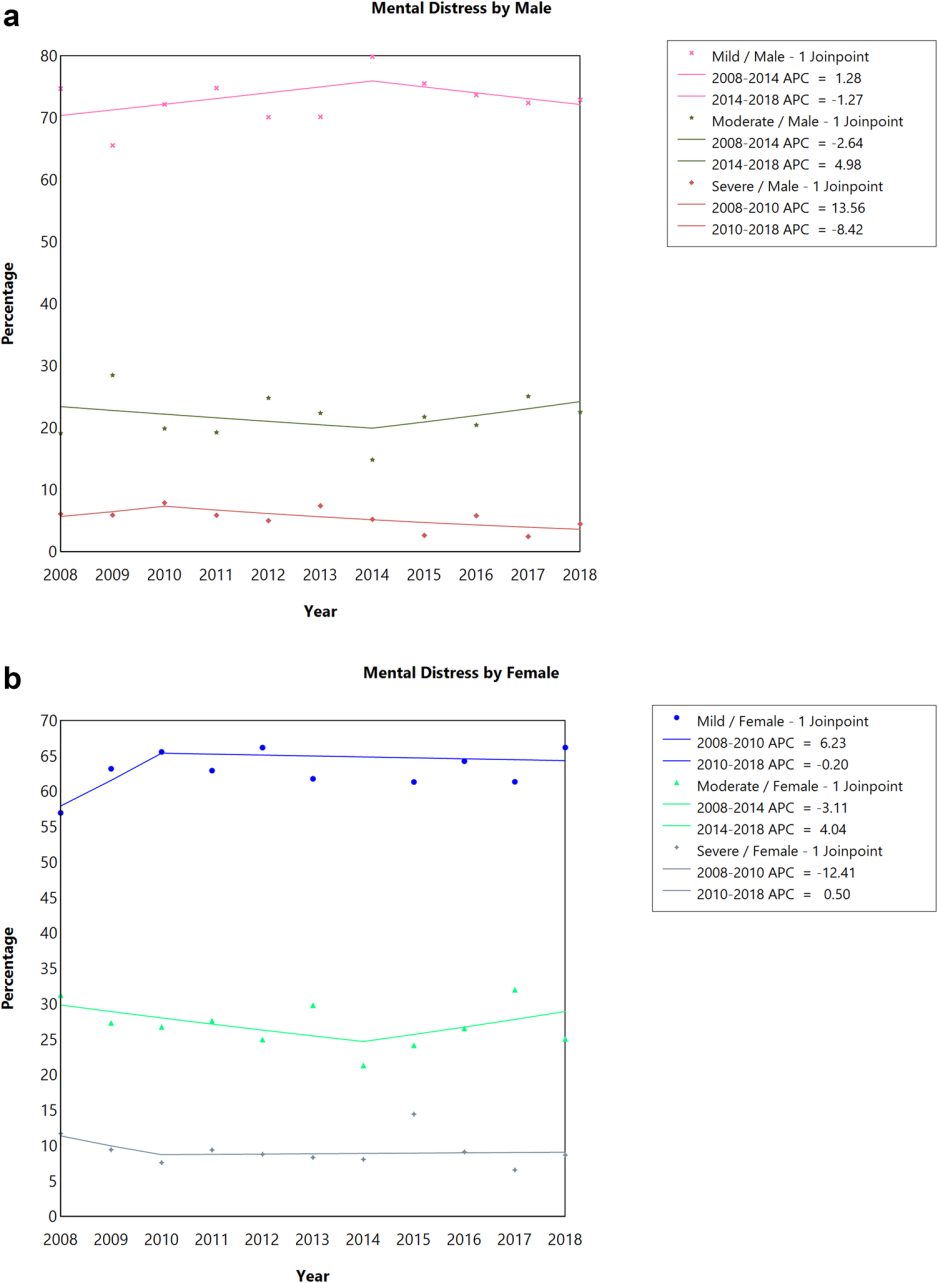
Joinpoint analysis of the prevalence of emotional distress among **a**) male and **b**) female cancer survivors diagnosed at childhood or adolescent and young adult, 2008–2018 National Health Interview Survey. APC, annual percentage change; ^*^P-value < 0.05 as test of significance. Data: 2008–2018 National Health Interview Survey

**Fig. 3:**
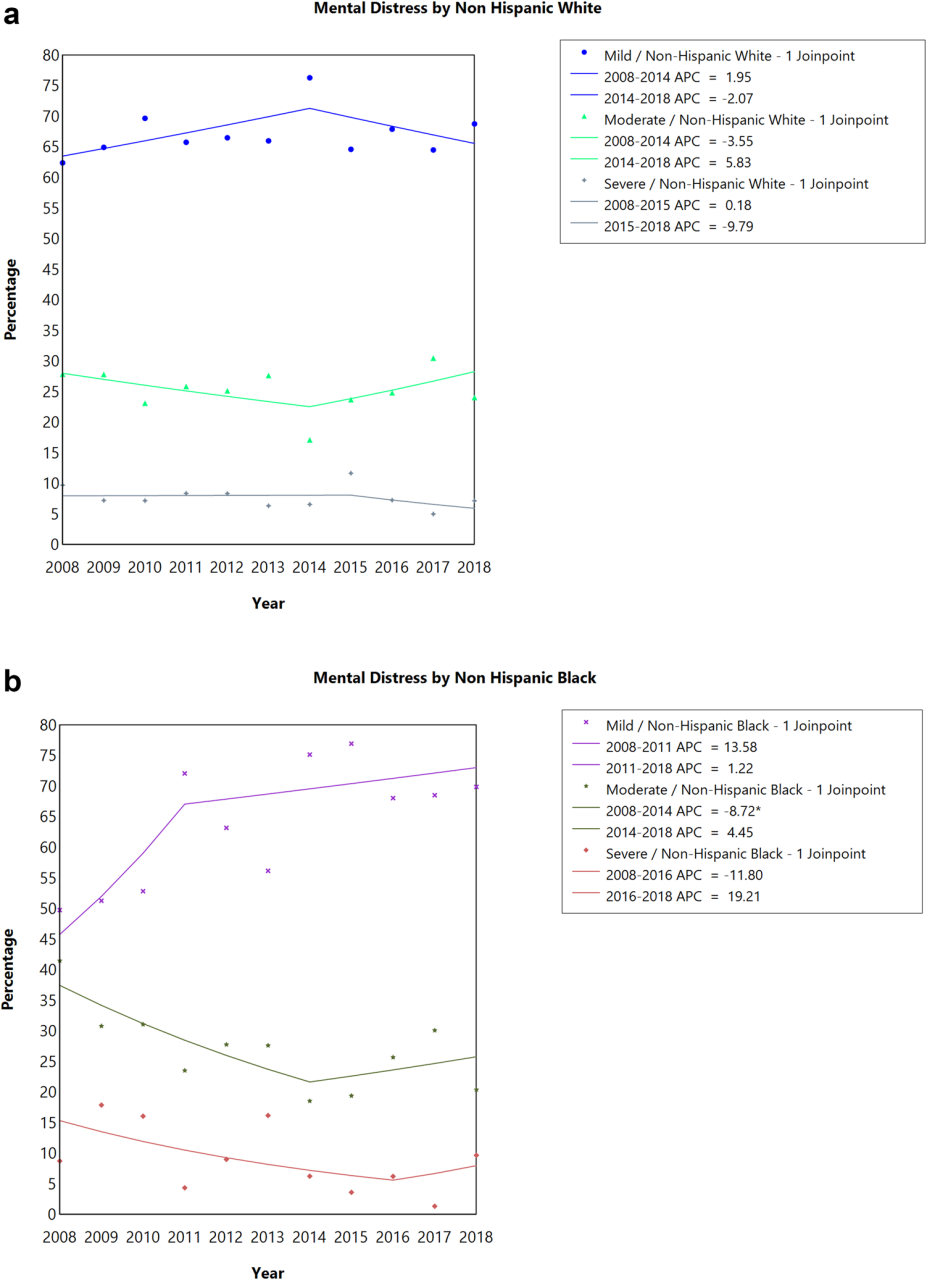

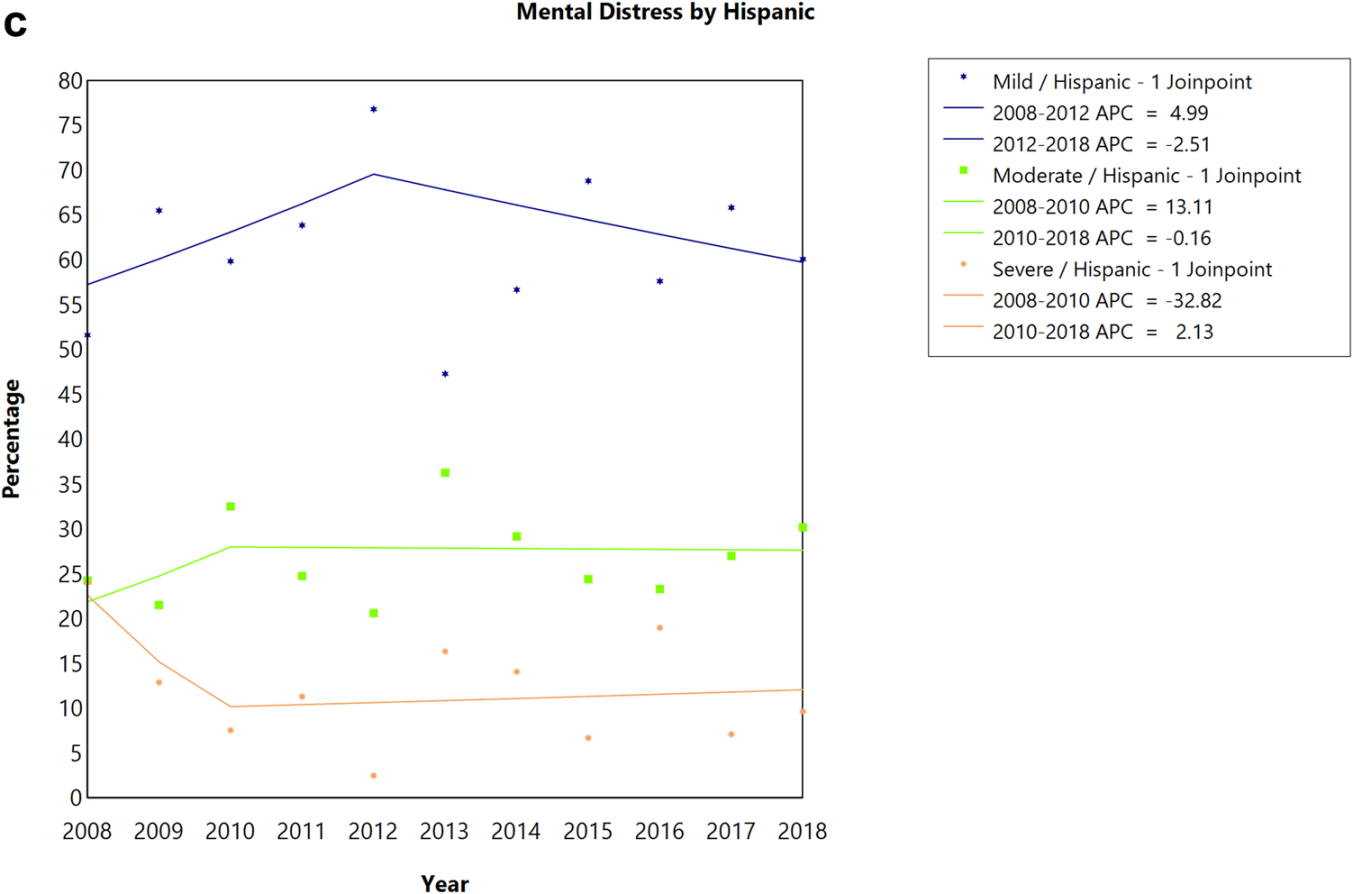
Joinpoint analysis of the prevalence of emotional distress among **a**) Non-Hispanic White, **b**) Non-Hispanic Black, and **c**) Hispanic cancer survivors diagnosed at childhood or adolescent and young adult, 2008–2018 National Health Interview Survey. APC, annual percentage change; ^*^P-value < 0.05 as test of significance. Data: 2008–2018 National Health Interview Survey

When stratified by sex, there was no statistically significant difference in trends in the proportion of individuals experiencing severe, moderate, or mild/no distress among both females and males CAYA survivors **(**Fig. 2A, B**, **Table 2**)**. There was no statistically significant difference in trends in the proportion of individuals experiencing severe, moderate, or mild/no distress for non-Hispanic Whites and Hispanics over the 10-year period **(**Fig. 3A, C**, **Table 2**)**. However, for non-Hispanic Blacks, the proportion of individuals experiencing moderate distress significantly decreased by 8.72% annually between 2008 and 2014 (APC = −8.72; 95% CI: −15.83 to −1.01, *P *= 0.033) and then increased by 4.45% annually between 2014 and 2018, although not statistically significant (Fig. 3B, Table 2). There was no statistically significant difference in trends in the proportion of individuals experiencing severe, moderate, or mild/no distress irrespective of the marital status of the respondent (Supplemental Fig. 1A-C, Table 2). There was no statistically significant difference in trends in the proportion of individuals experiencing severe, moderate, or mild/no distress among respondents who visited or did not visit a mental health professional in the past 12 months (Supplemental Fig. 2A-B, Table 2).

## Discussion

Our study aimed to assess trends in emotional distress among the CAYA cancer survivors. To our knowledge, this is the first study investigating trends in emotional distress over time among survivors of CAYA cancers. Our results reported that overall trends in emotional distress have not changed significantly over the 2008–2018 decade. When stratified by gender, race/ethnicity, marital status, and visit to mental health professional within the past year, we did not observe any significant changes in the trend of emotional distress except in non-Hispanic Blacks. Non-Hispanic Blacks experiencing moderate distress had a significantly decreased trend between 2008 and 2014. Though traditionally, non-Hispanic Blacks report lower mental health due to protective factors like strong social support, religious hope, and unique cultural coping mechanisms; it is difficult to explain this finding as we could not access changes in those factors in the same timeframe. Future studies should examine trends in protective factors and mental health to help explain this relationship. If distress levels remain unchanged, there may be serious long-term implications on the quality of life and healthcare outcomes of survivors. Persistent emotional distress is associated with poor adherence to treatment, worse overall outcomes, and significant financial burdens [21], all of which highlight the need for support for this population.

Although not statistically significant, our analyses revealed a slight increase in moderate emotional distress but a slight decrease in mild emotional distress from 2014 to 2018. The proportion of CAYA cancer survivors with severe distress was relatively stable through the study period. While our findings did not report a significant increase in distress over time, the consistency of moderate and severe distress levels over the years suggests that emotional/psychological distress remains an unresolved issue for many CAYA survivors, consistent with literature suggesting that higher levels of emotional distress among cancer survivors compared to the general population [12, 22]. Evidence indicates that a history of mental illness is associated with poorer outcomes [21], which could explain some of the persistent distress observed in our data. Other potential explanations could be the long-term psychosocial impacts of cancer treatment or the growing stigma of mental health challenges in recent years and navigating adult responsibilities while coping with the long-term effects of cancer may also contribute to sustained distress among survivors [23, 24]. The challenges of employment, education, and relationships are further complicated for survivors diagnosed as AYAs, underscoring the importance of specialized support services [25]. This situation is compounded by the lack of treatment protocols specifically designed for survivors diagnosed as children or AYAs and the limited availability of clinical trials focused on this age group [26]. Our findings highlight the persistent psychological burden faced by cancer survivors, emphasizing the need for continued screening and support, regardless of emotional distress severity.

Our analysis of emotional distress trends stratified by gender, race/ethnicity, marital status, and visit to mental health professionals was not statistically significant. This finding indicates that psychological distress is a universal challenge among cancer survivors, transcending gender, race/ethnicity, and even access to mental health professionals. The trend among these groups has not changed significantly over the years, suggesting that emotional distress remains a consistent issue across all demographics. This finding diverges from prior cross-sectional studies that have consistently shown greater emotional distress among female survivors, as well as elevated distress among ethnic minorities and individuals with limited access to mental health services [27–29]. For example, cross-sectional studies have shown that women, particularly adolescent females, tend to experience higher rates of depression and anxiety compared to their male counterparts, which reemphasizes the complexity of CAYA cancer survivor subgroup needs [30, 31]. Social and psychological stressors such as infertility and the long-term sequelae of cancer treatment may exacerbate these issues among female survivors [32, 33]. This could indicate that current interventions are not sufficiently tailored to address the complex needs of CAYA survivor subgroups [30].

Interestingly, despite an increase in visits to mental health professionals, trends in emotional distress levels among CAYA survivors remained stable. A possible explanation for this could be the persistent stigma surrounding mental health, particularly in the previous two decades. Research indicates that while attitudes toward mental health have improved over the years, stigma still lingers, especially in certain populations, which might prevent survivors from fully benefiting from mental health services [24, 34]. This finding suggests that even though mental health services are being accessed, current approaches may not be sufficient to address emotional distress in CAYA survivors. These unmet needs may contribute to persistently heightened emotional distress, as suggested by previous literature [31]. Pre-existing mental health conditions, which were not captured in our data, may also play a role.

### Limitations

Our findings should be interpreted in the context of several limitations. First, the use of self-reported data regarding emotional distress introduces the potential for recall and social desirability biases. Additionally, a small sample size limited the examination of trends in emotional distress by cancer type to assess if distress differed by cancer a survivor was diagnosed with. Lastly, due to the cross-sectional design, we fail to comment on the causal association of the study.

## Conclusion

Our study provides a novel examination of emotional distress trends among CAYA cancer survivors over a decade. While overall distress levels have remained relatively stable, the consistent presence of moderate and severe distress underscores the need for continued mental health support and tailored interventions. Understanding the nuanced needs of this survivor population and its subgroups is critical in improving their quality of life and long-term outcomes.

## Supplementary Information

Below is the link to the electronic supplementary material.

ESM 1 Supplementary Material 1 (PNG 99.4 KB)

High Resolution Image (TIFF 94.4 KB)

ESM 2 Supplementary Material 2 (PNG 111 KB)

High Resolution Image (TIFF 100 KB)

ESM 3 Supplementary Material 3 (PNG 109 KB)

High Resolution Image (TIFF 103 KB)

ESM 4 Supplementary Material 4 (PNG 99.1 KB)

High Resolution Image (TIFF 95.4 KB)

ESM 5 Supplementary Material 5 (PNG 100 KB)

High Resolution Image (TIFF 95.0 KB)

## Data Availability

The data used in this study are publicly available and can be accessed from NHIS at https://www.cdc.gov/nchs/nhis/documentation/index.html.
