# Supplementary figures and images for: Trends in emotional distress among childhood, adolescent, and young adult (CAYA) cancer survivors: A decade-long study
Source: Support Care Cancer. 2026 Mar 23;34(4):352. doi: 10.1007/s00520-026-10580-7 (PMC13006460; doi:10.1007/s00520-026-10580-7)

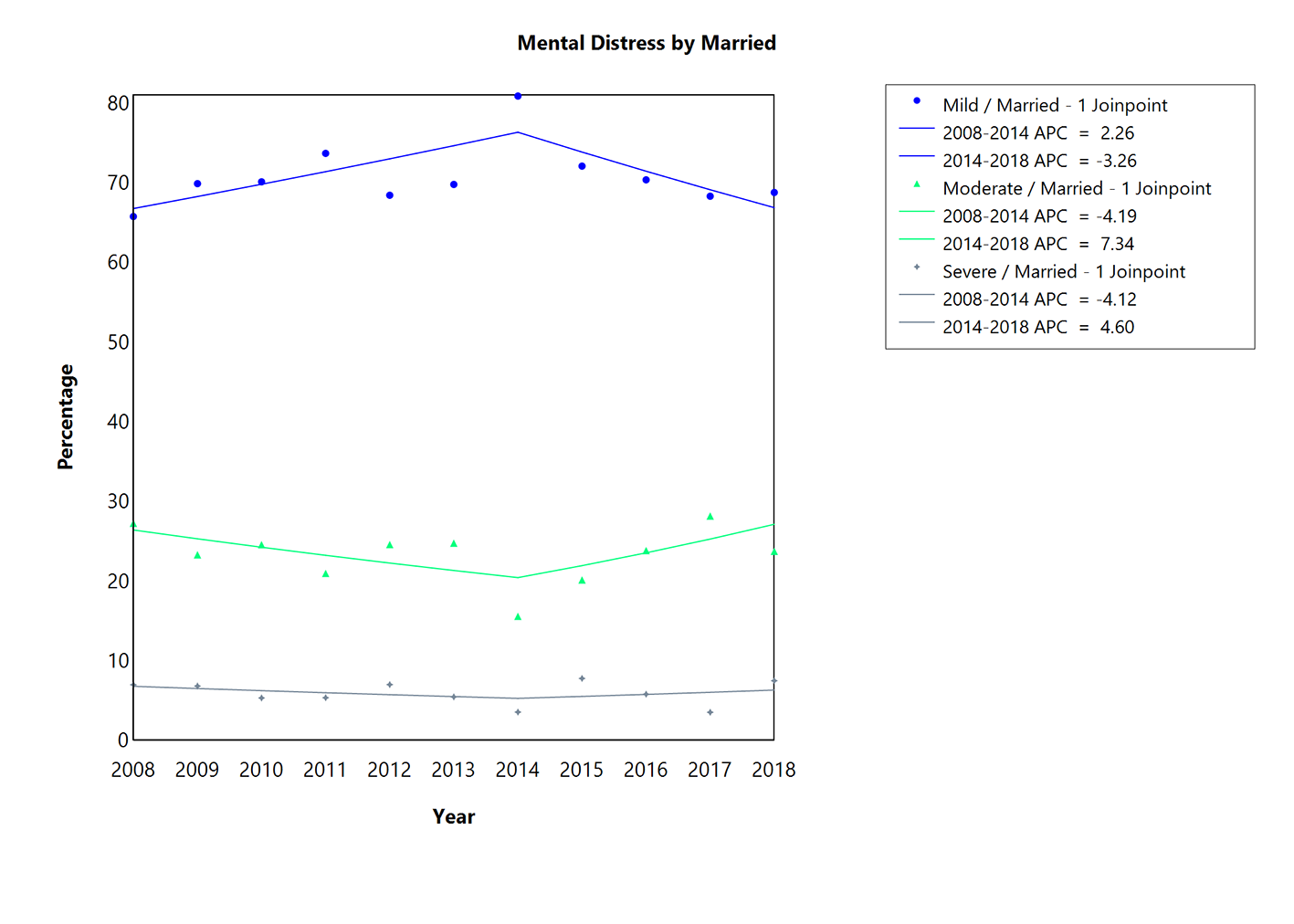

Supplement: Supplementary file 1 — Supplementary Material 1 (PNG 99.4 KB) [file 520_2026_10580_Fig4_ESM.png]

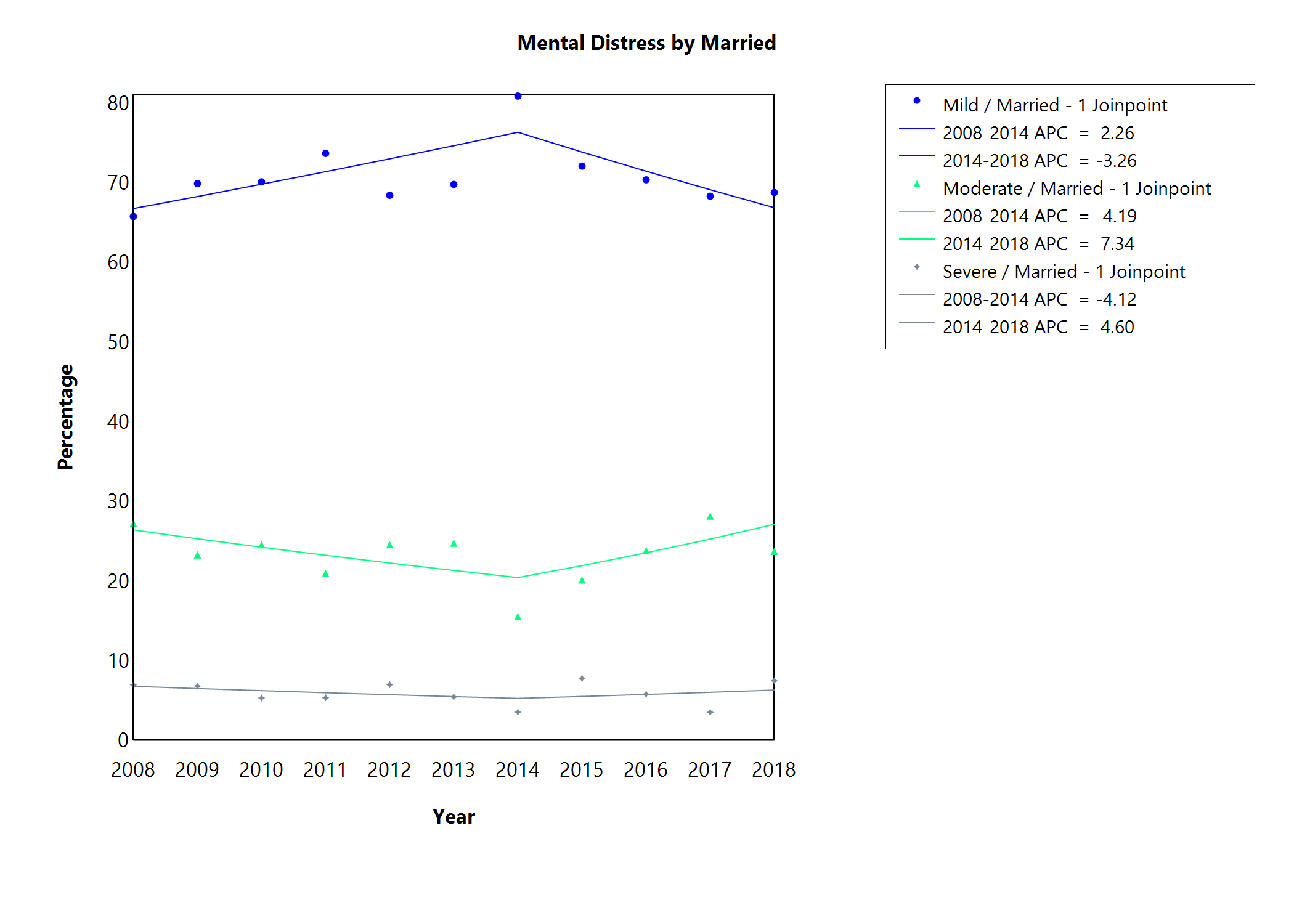

Supplement: Supplementary file 2 — High Resolution Image (TIFF 94.4 KB) [file 520_2026_10580_MOESM1_ESM.tiff]

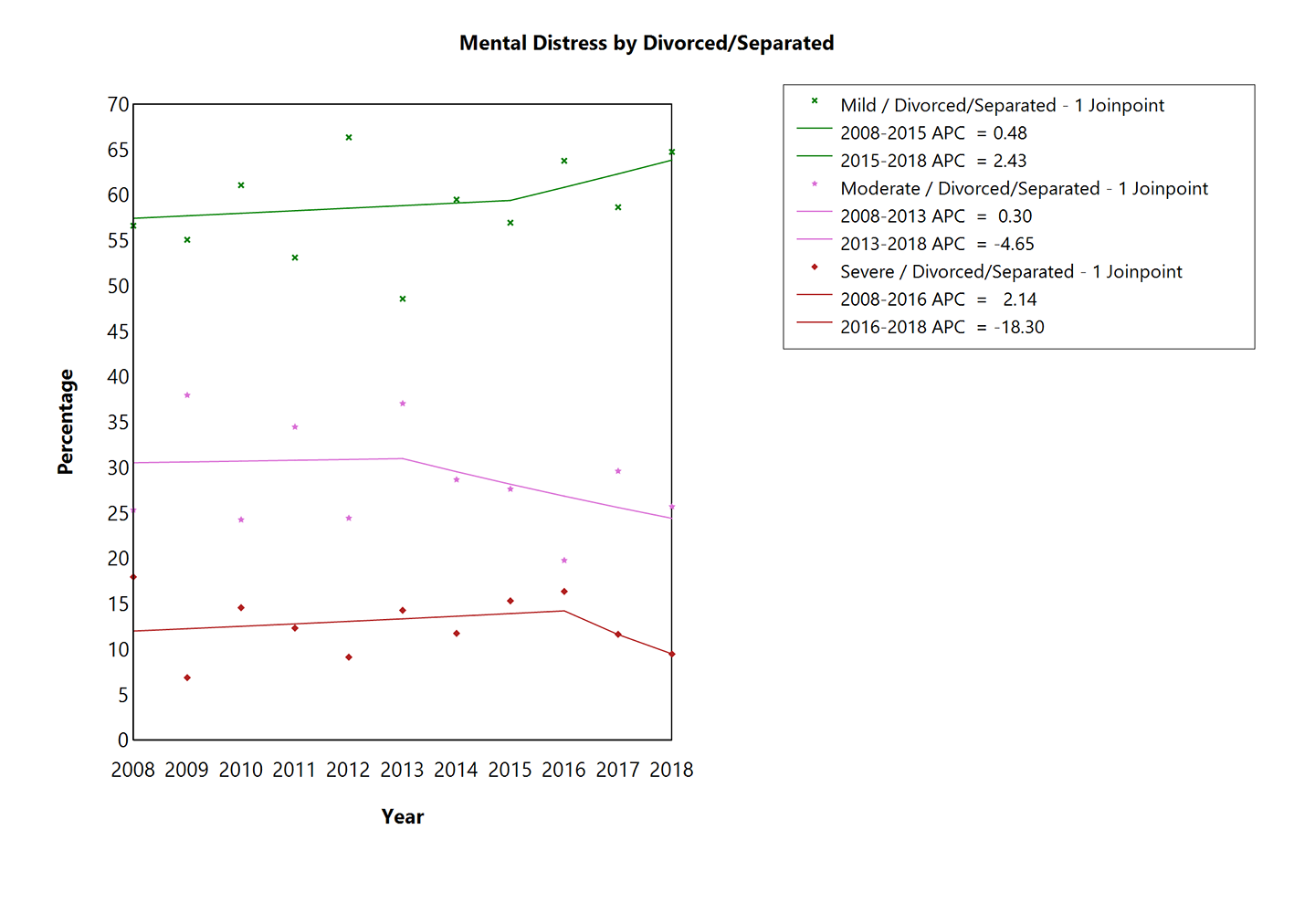

Supplement: Supplementary file 3 — Supplementary Material 2 (PNG 111 KB) [file 520_2026_10580_Fig5_ESM.png]

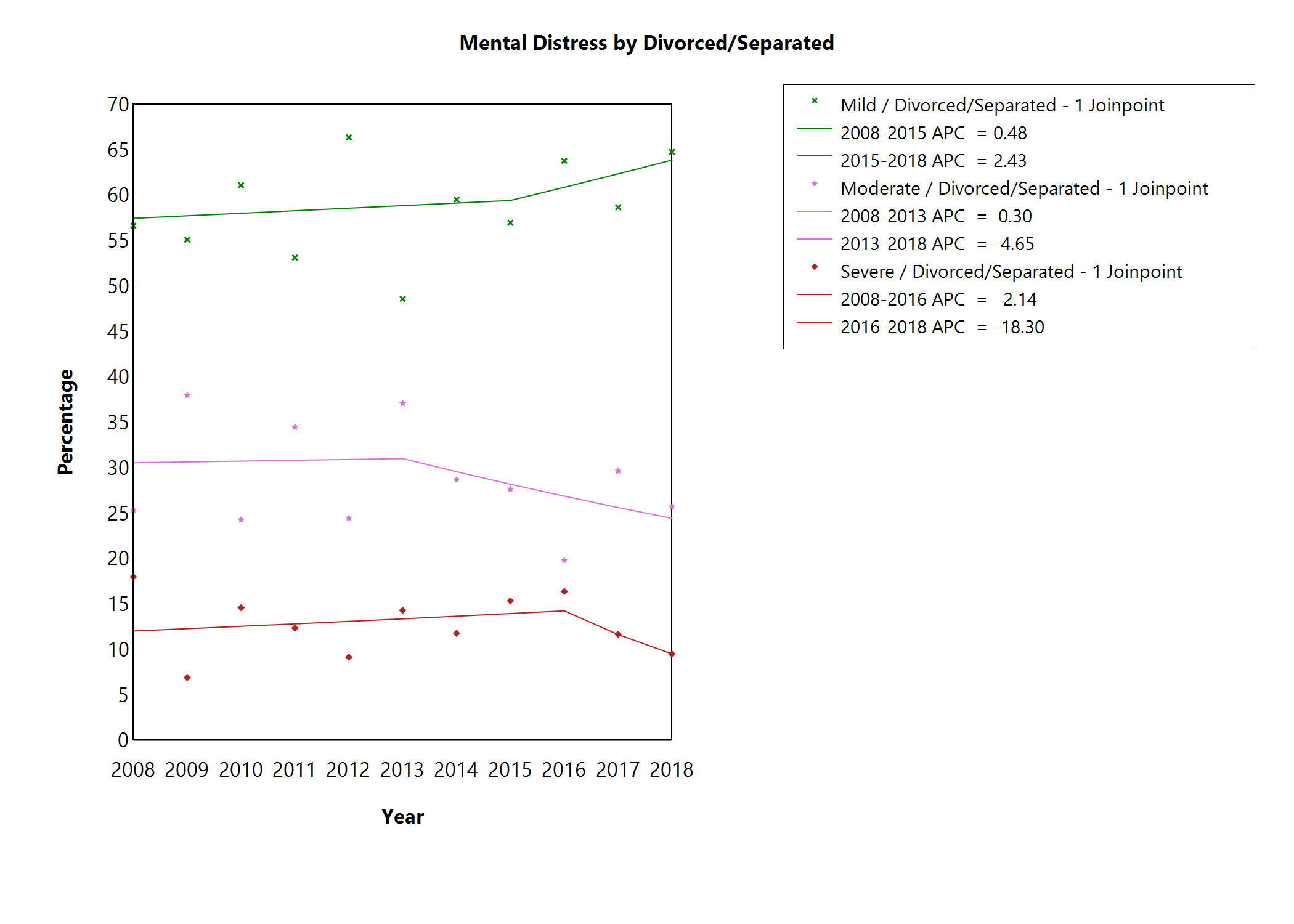

Supplement: Supplementary file 4 — High Resolution Image (TIFF 100 KB) [file 520_2026_10580_MOESM2_ESM.tiff]

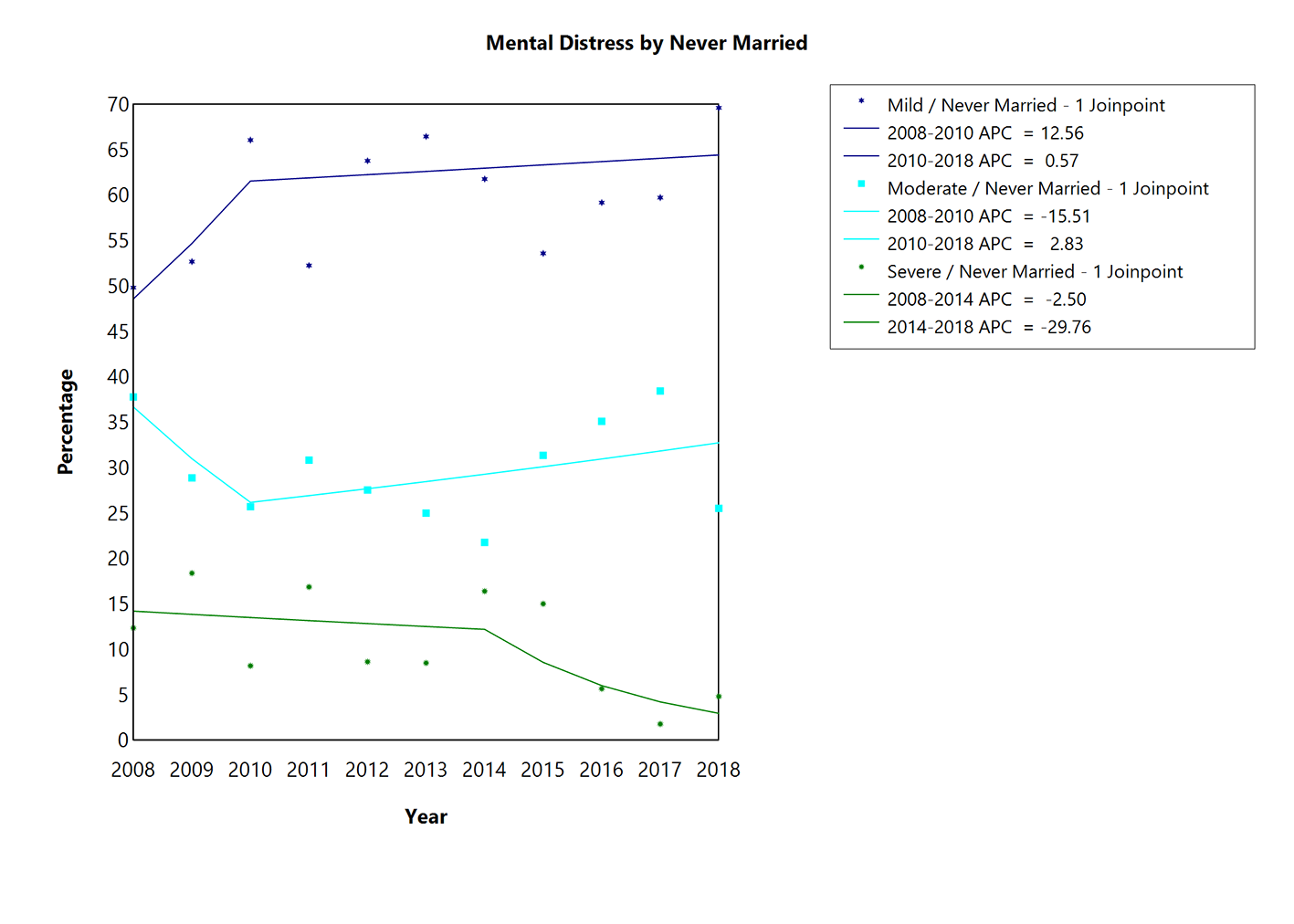

Supplement: Supplementary file 5 — Supplementary Material 3 (PNG 109 KB) [file 520_2026_10580_Fig6_ESM.png]

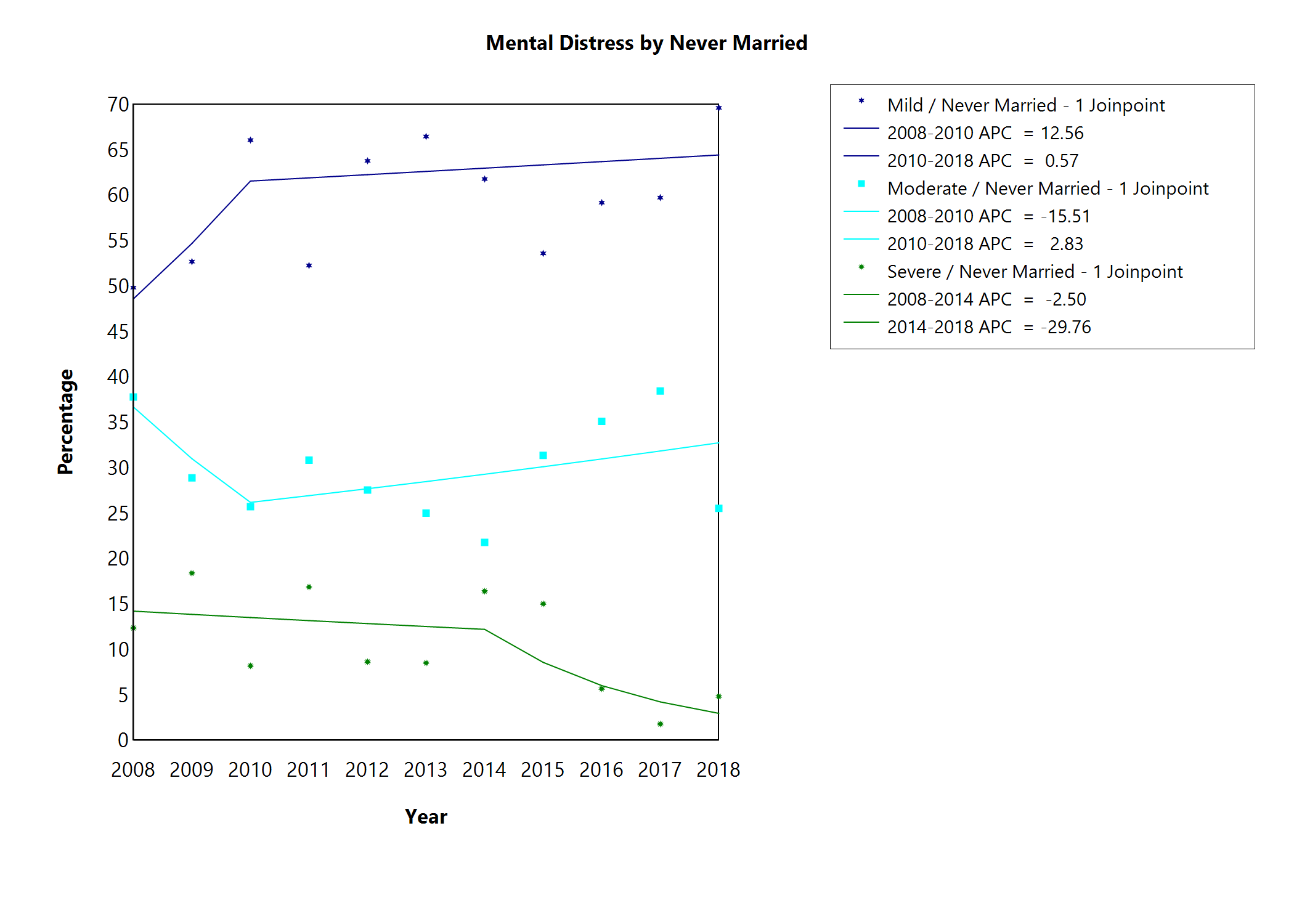

Supplement: Supplementary file 6 — High Resolution Image (TIFF 103 KB) [file 520_2026_10580_MOESM3_ESM.tiff]

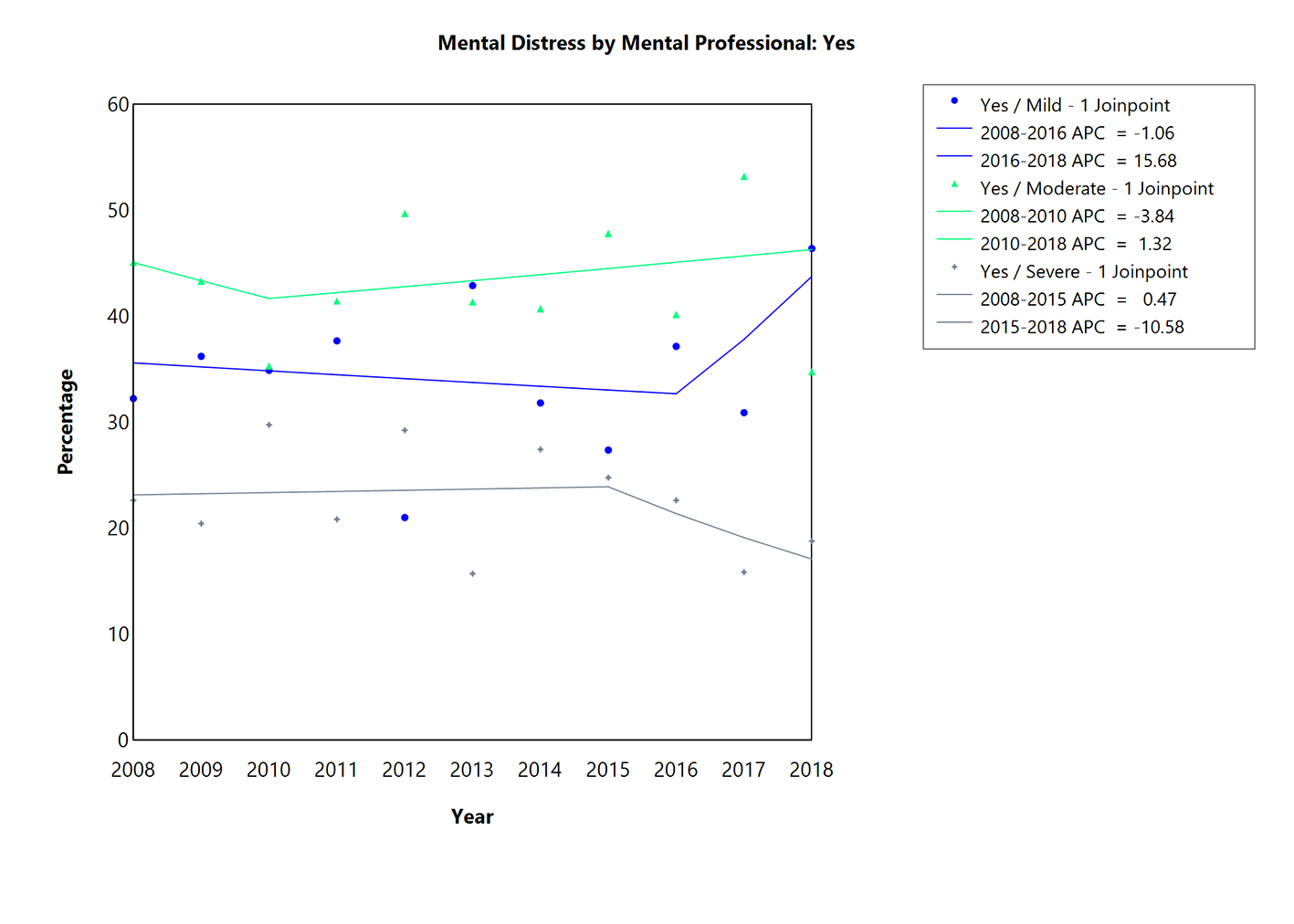

Supplement: Supplementary file 7 — Supplementary Material 4 (PNG 99.1 KB) [file 520_2026_10580_Fig7_ESM.png]

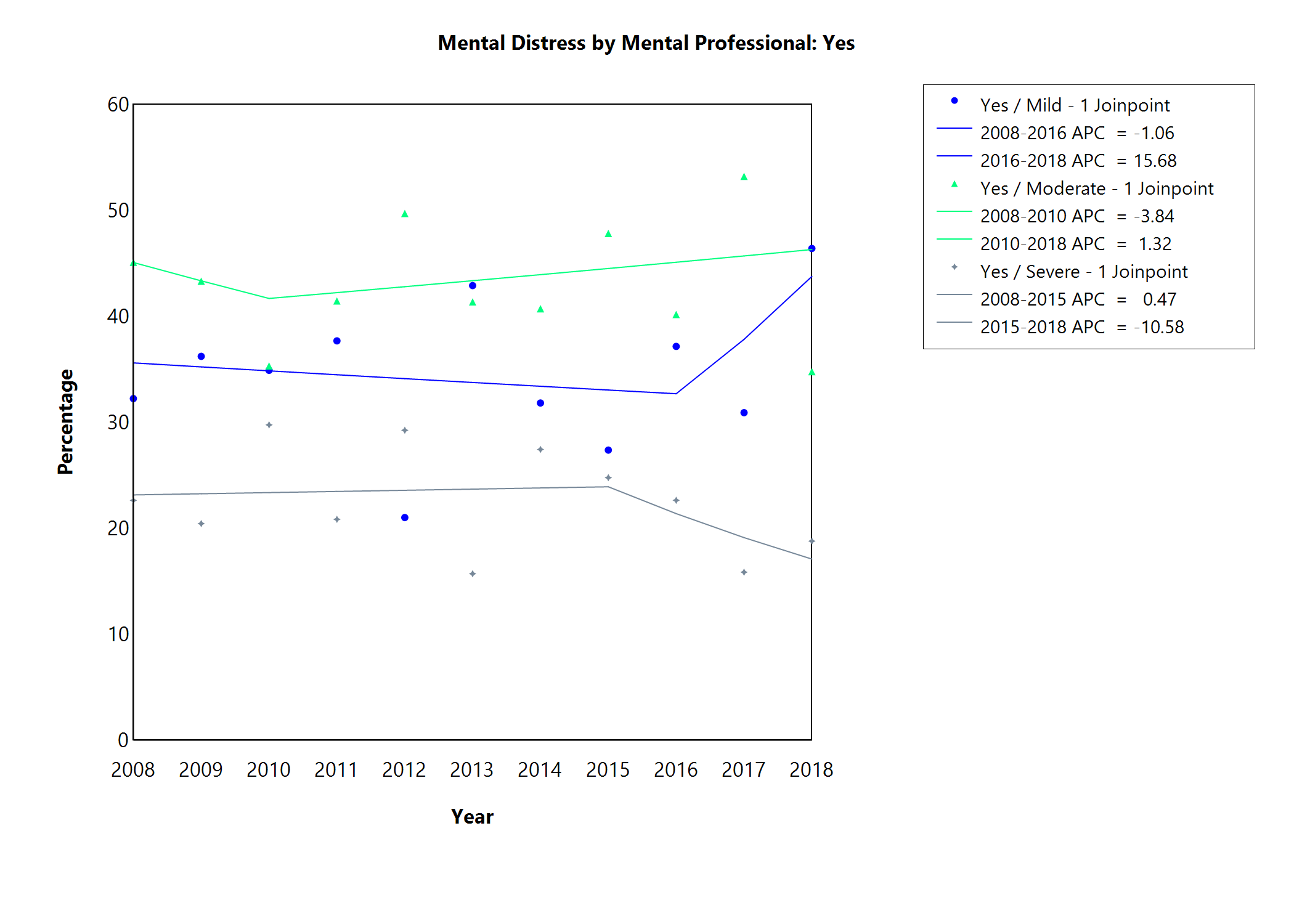

Supplement: Supplementary file 8 — High Resolution Image (TIFF 95.4 KB) [file 520_2026_10580_MOESM4_ESM.tiff]

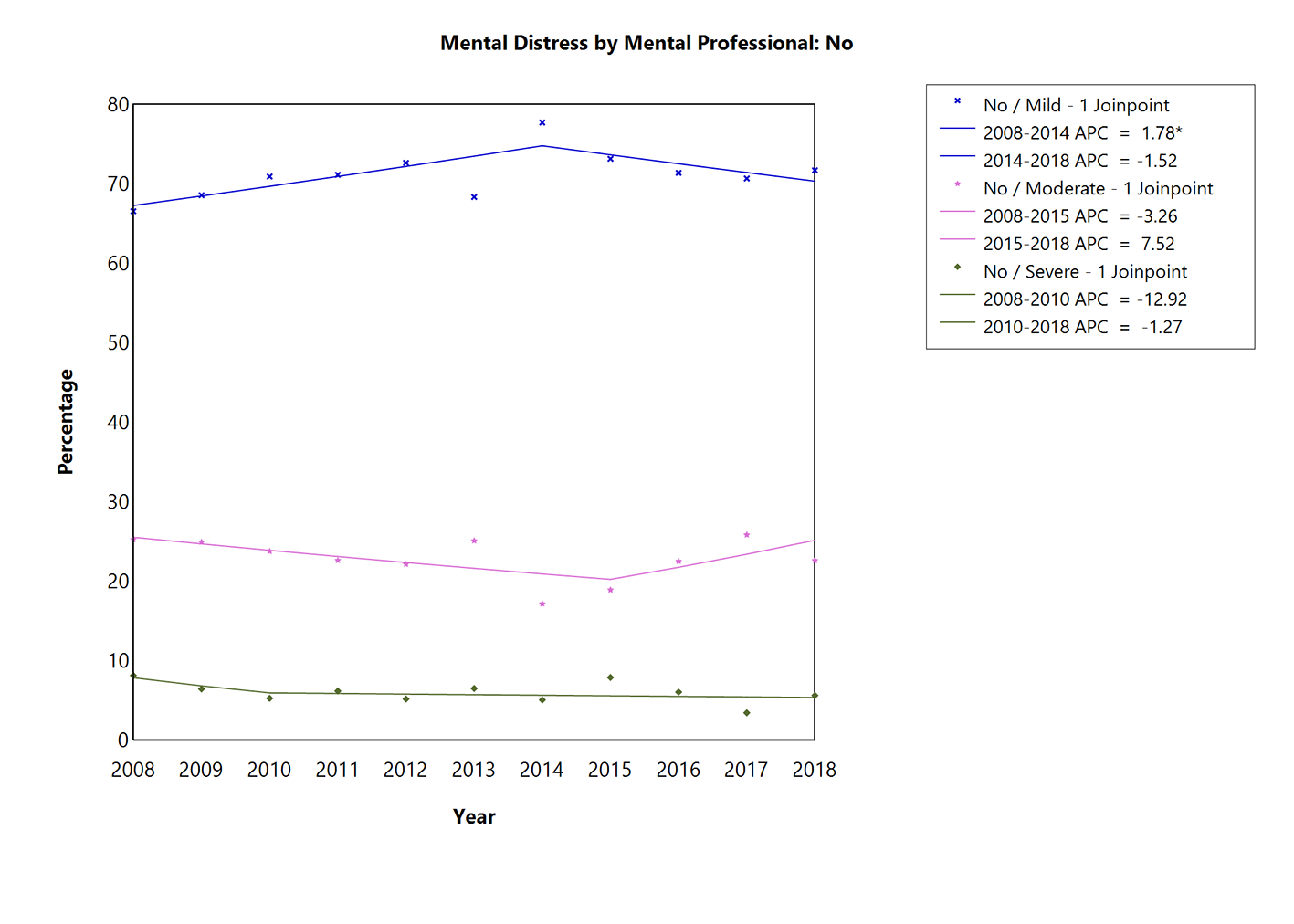

Supplement: Supplementary file 9 — Supplementary Material 5 (PNG 100 KB) [file 520_2026_10580_Fig8_ESM.png]

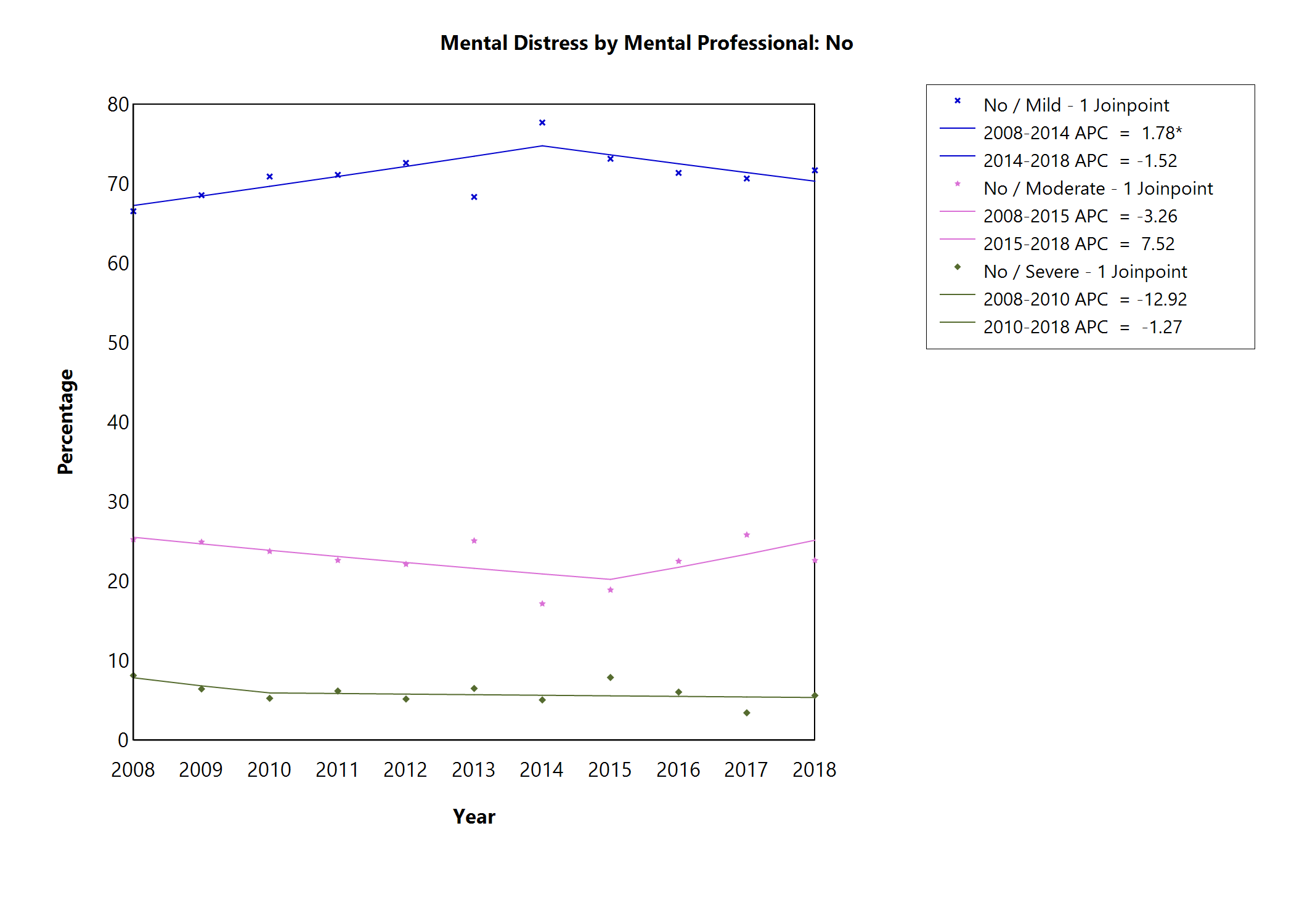

Supplement: Supplementary file 10 — High Resolution Image (TIFF 95.0 KB) [file 520_2026_10580_MOESM5_ESM.tiff]
